# Microvascular comparison in younger and older patients with retinal vein occlusion analyzed by OCT angiography

**DOI:** 10.1186/s12886-021-01931-5

**Published:** 2021-04-05

**Authors:** Panpan Ye, Tiepei Zhu, Fang Zheng, Min Zhou, Xiaoyun Fang, Ke Yao

**Affiliations:** 1 Eye Center, Second Affiliated Hospital, School of Medicine, Zhejiang University, No.88, Jiefang Road, 310009 Hangzhou, China; 2 The Institute of Translational Medicine, Zhejiang University, Hangzhou, China

**Keywords:** Retinal vein occlusion, Anti-VEGF treatment, OCT angiography, Retinal vasculature

## Abstract

**Background:**

To compare changes in retinal microvasculature of young and elderly patients with retinal vein occlusion (RVO) after anti-VEGF treatment.

**Methods:**

RVO patients who underwent anti-VEGF treatment were retrospectively reviewed and categorized into two groups based on age. The OCT angiography images were obtained during each visit. Best corrected visual acuity (BCVA), vessel density (VD) and foveal avascular zone (FAZ) were measured and compared between the two groups. Vision improvements and retinal microvasculature changes were also correlated.

**Results:**

Twenty patients with 20 eyes were enrolled in the younger group and 46 patients with 46 eyes were enrolled in the older group. Younger patients demonstrated better BCVA, higher VD and smaller FAZ than older patients at 12 months after the first anti-VEGF treatment. The improvement of VD was observed only in the younger group. A positive correlation between vision improvement and VD increase was noted.

**Conclusions:**

Young patients with RVO can achieve rapid rehabilitation of deep retinal vasculature which lead to a better visual outcome.

## Background

Retinal vein occlusion is one of the most common retinal vascular diseases influencing approximately 16 million patients worldwide [1]. It usually occurs in elder people, and the predisposing factors include hypertension, hyperglycemia, hyperlipidemia, smoking, and alcohol consumption. Patients often present with venous tortuosity, venous distention, and retinal hemorrhage. Recurrent macular edema, retinal ischemia, and neovascularization are the major reasons for vision loss. The pathological changes of RVO can persist for several months or even years. As the pace of daily life and workplace expectations have accelerated, more young people are suffering from RVO than previous decades. Compared with elderly patients, the risk factors of RVO in young patients is untraditional, though it may be correlated with inflammation [2].

Optical coherence tomography angiography (OCTA), a new technology for detecting retinal microvascular architecture, displays high-resolution images of retina and choroid in separated layers. Recently, an upgraded OCTA software program with a projection artifact removal (PAR) function has been applied in clinical settings [3]. With this advanced technique, the deep capillary structure can be clearly captured and accurately measured, including foveal avascular zone (FAZ) area, vessel density (VD), vessel length density, and size of non-perfusion area [4]. In RVO patients, OCTA can be used to track the change of retinal microvasculature and assess the rehabilitation of blood flow. In this study, we aimed to evaluate the impact of aging on the retinal capillaries reconstruction using OCTA, as well as the correlation of these changes with visual outcomes in patients with RVO.

## Methods

Patients diagnosed with RVO from September 2017 to October 2019 were enrolled in a retrospective study at Eye Center, Second Affiliated Hospital of Zhejiang University. The Institutional Review Board of the Second Affiliated Hospital of Zhejiang University (No.2019 − 406) approved the study. The study was performed in accordance with the tenets of the Declaration of Helsinki and compliant with the Health Insurance Portability and Accountability Act of 1996. The need for written informed consent was waived by the Institutional Review Board of the Second Affiliated Hospital of Zhejiang University (No.2019 − 406) because of the retrospective design and the use of de-identified patient data.

Patients of macular involved RVO, either central RVO (CRVO), or branch RVO (BRVO), with at least 12-month follow-up and OCTA obtained in each visit were selected. The OCTA images were acquired by AngioVue OCTA system (version 2018.0.01.14, OptovueRTVue XR 100; AVANTI, Inc). Macular angiogram was detected by a 3 × 3 mm scan centered on the fovea. This instrument uses a split-spectrum amplitude decorrelation angiography (SSADA) software algorithm, acquiring 70,000 A-scans/s to compose OCT-A volumes consisting of 304 × 304 A-scans with two consecutive B-scans captured at each fixed position. Retinal layer segmentation was set as reported previously [5]. Manual correction of B-scans were performed if any layer segmentation errors were identified. The projection artifact in the deep capillary plexus (DCP) was automatically eliminated by the AngioVue software (version 2018.0.01.14). Parameters including whole 3 × 3 mm image and parafovea VD of the superficial capillary plexus (SCP) and DCP, size and perimeter of FAZ, as well as central retinal thickness (CRT) were recorded for all subjects.

The exclusion criteria include previous retinal surgery, ocular trauma, coexistence of other retina disorders such as diabetic retinopathy, pathologic myopia or age-related macular degeneration. Eyes that could not be assessed by OCTA due to media opacity, significant eye movements, or segmentation failure arising from severe macular edema were also excluded. Patients were categorized into younger group (50 years or younger at RVO onset) and older group (more than 50 years at RVO onset). All patients underwent a comprehensive routine ophthalmic examination including best-corrected visual acuity (BCVA), intraocular pressure (IOP), fundus photography, spectral domain OCT (Spectralis Heidelberg Engineering, Heidelberg, Germany), and/or fundus fluorescein angiography (FFA). Patients were examined for traditional risk factors. Younger patients without traditional risk factors underwent additional laboratory testing for possible etiology, including coagulation function, protein C and protein S, rheumatoid factor, anticardiolipin antibody, lupus anticoagulant, factor V Leiden, lupus anticoagulant, homocysteine, and cryoglobulins.

Intravitreal injections of the anti-VEGF agents (either ranibizumab or conbercept) were used to treat macular edema for RVO. The patients received an initial intravitreal anti-VEGF injection followed by a *pro re nata* (PRN) regimen with monthly monitoring. Retreatment with anti-VEGF injection was considered if the recurrence of macular edema detected by SD-OCT exceeding 300 μm CRT. Scatter laser photocoagulation was applied for retinal neovascularization and/or nonperfused areas (NPAs) more than 5 disk area (DA) for eyes with BRVO and NPAs more than 10 DA for eyes with CRVO.

SPSS for Windows version 17.0 (SPSS Inc., Chicago, IL) was used for statistical analysis. A *P* < 0.05 was considered statistically significant. Quantitative variables were presented as mean ± SD or median (interquartile range). Categorical variables were expressed as values and percentages. Vision improvement rate was used to evaluate the degree of vision recovery. Student’s *t* test, Mann-Whitney test, and Chi-square test were used to compare variables. Pearson’s *r* was used to summarize the strength of the correlations.

## Results

Sixty-six RVO patients with 66 eyes who were followed for at least 12 months were enrolled. Of these patients, 20 were 50 years old and younger (38.3 ± 7.7, range:22–50), and 46 were older than 50 years old (63.0 ± 6.7, range:52–85). There was no significant difference in the distribution of sex or eye laterality. There was a trend toward better BCVA at the initial visit in younger patients than older patients (logMAR 0.63 ± 0.44 vs. 0.81 ± 0.36, *P* = 0.078). Of these younger patients, one used oral contraceptives, one was diagnosed with ankylosing spondylitis, two had increased homocysteine levels, and one had severe anemia. One young patient had recurrent retinal and brain vascular occlusion, and the coagulation panel sequence result showed coagulation factor XII deficiency. All patients received one intravitreal injection of anti-VEGF as the initial treatment. The mean number of injections in 12 months was 3.2 ± 2.1 (range:1–7) in the younger group and 3.8 ± 2.2 (range:1–9) in the older group (*P* = 0.320). Scatter laser coagulation was performed in 8 out of 20 eyes in the younger group and in 18 out of 46 eyes in the older group, which was not significantly different (*P* = 0.947). All general information of patients is summarized in Table 1.

**Table 1:** Patient characteristics

	Younger group (*n* = 20)	Older group (*n* = 46)	*P*
Age	38.3 ± 7.7	63.0 ± 6.7	< 0.001
Male	12	16	0.057
Initial BCVA (LogMAR)	0.63 ± 0.44	0.81 ± 0.36	0.078
Right Eye	9	24	0.592
CRVO/BRVO	9/11	21/25	0.961
Risk Factors			
Hypertension	3	15	0.140
Diabetes	2	6	0.728
Hyperlipidemia	2	10	0.256
Glaucoma	4	5	0.321
Injection Number in 12 months	3.2 ± 2.1	3.8 ± 2.2	0.320
Case Number with laser coagulation	8	18	0.947

*BCVA* best corrected visual acuity; *CRVO* central retinal vein occlusion; *BRVO* branch retinal vein occlusion

**Fig. 1:**
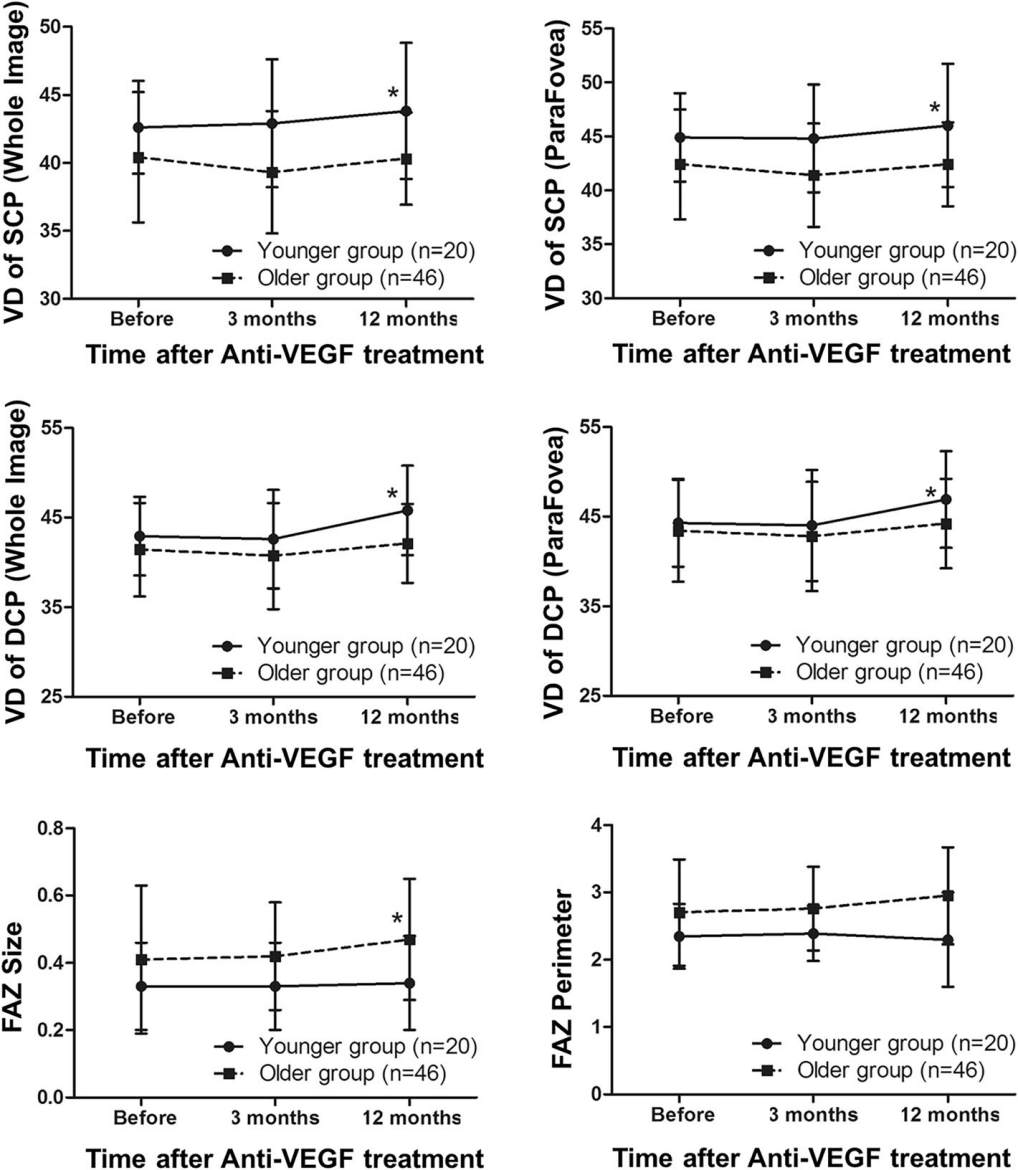
The comparison of vessel density and fovea avascular zone change after anti-VEGF treatment. VD: vessel density; SCP: superficial capillary plexus; DCP: deep capillary plexus; FAZ: foveal avascular zone. *P < 0.05

**Fig. 2:**
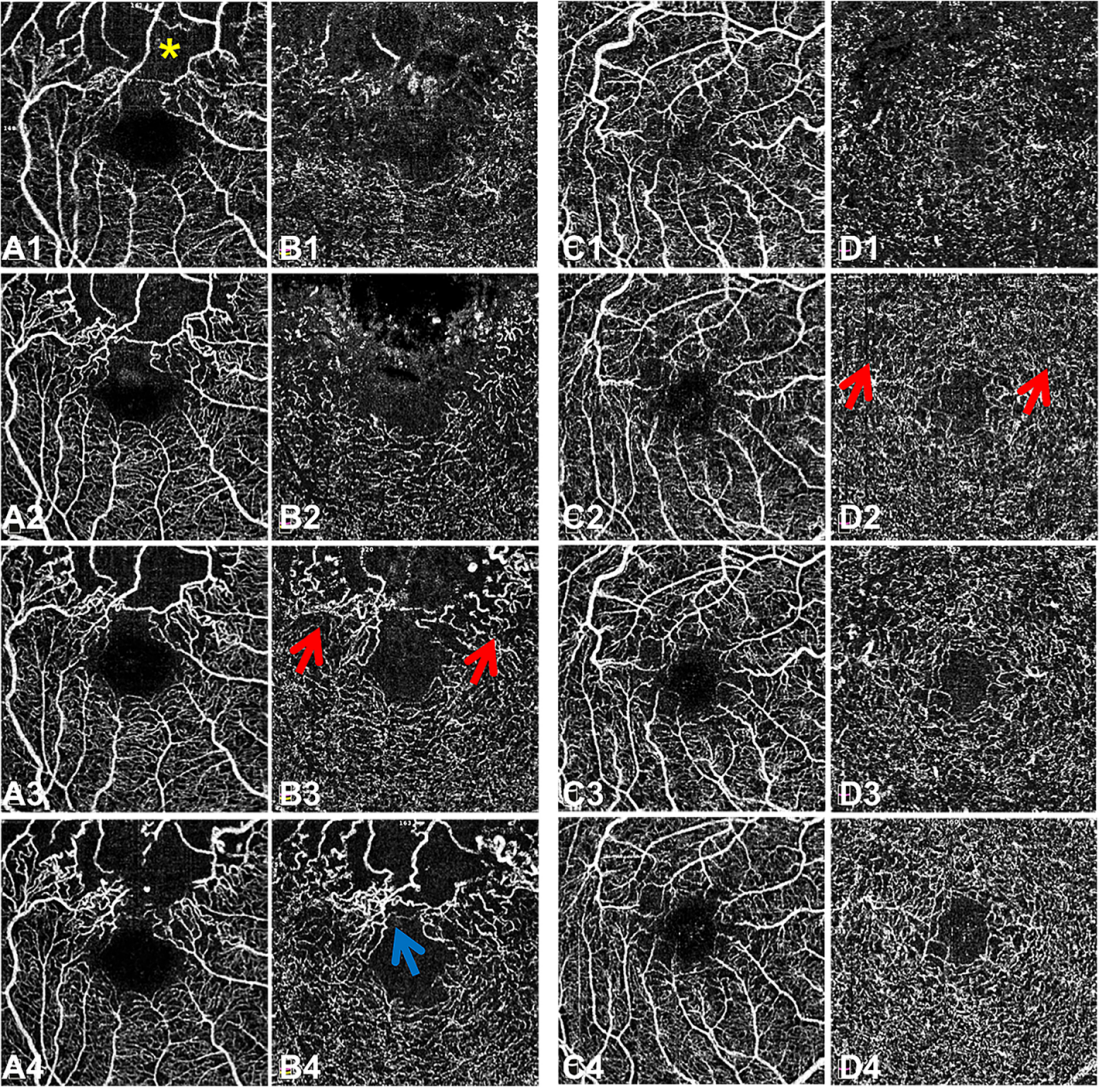
OCTA images of the superficial and deep capillary plexus in a 64-year-old patient (**a** and **b**) and a 25-year-old patient (**c** and **d**). (**A1**, **A2**, **A3**, **A4**) The OCTA images of superficial capillary plexus of the 64-year-old patient at baseline, 1 month, 3 months and 12 months after the first anti-VEGF injection showing the enlargement of FAZ and disappearance of a branch retina capillary (yellow asterisk). (**B1**, **B2**, **B3**, **B4**) The OCTA images of deep capillary plexus of the 64-year-old patient at baseline, 1 month, 3 months and 12 months after the first anti-VEGF injection presenting the reconstruction of some retinal vasculature (red arrows) and diving of the superficial retina vessels into the deep layer due to retina atrophy (blue arrow). (**C1**, **C2**, **C3**, **C4**) The OCTA images of superficial capillary plexus of the 25-year-old patient at baseline, 1 month, 3 months and 12 months after the first anti-VEGF injection showing decrease of vascular tortuosity around fovea. (**D1**, **D2**, **D3**, **D4**) The OCTA images of deep capillary plexus of the 25-year-old patient at baseline, 1 month, 3 months and 12 months after the first anti-VEGF injection presenting the reconstruction of retinal vasculature (red arrows)

There were no significant differences in VD of SCP and DCP or size and perimeter of FAZ at the first visit between the two groups (Table 2). Younger patients showed increased macular VD in both superficial and deep retina circulation and smaller FAZ after anti-VEGF treatment (Table 2). Microvascular improvement by anti-VEGF treatment was achieved only in the younger group, with significant difference in the increase of VD in DCP between two groups (Fig. 1; Table 3). By contrast, the FAZ size increased in the older group after treatment (Fig. 1), which can also be observed in the example shown in Fig. 2. A significant improvement of BCVA after treatment was noted in both younger and older group (both *P* < 0.001). Compared with the older group, the younger group showed more increase of BCVA (*P* = 0.007) and significantly better vision (*P* = 0.003) after treatment vision (Table 4). Significant positive correlation between VD increase and vision improvement rate after anti-VEGF treatment was found only in DCP (Table 5).

**Table 2:** Microvascular parameters measurements before and after VEGF treatment

	Younger group (*n* = 20)	Older group (*n* = 46)	*P*
VD of SCP (Whole Image)			
Baseline	42.6 ± 3.4	40.4 ± 4.8	0.112
3 months after treatment	42.9 ± 4.7	39.3 ± 4.5	0.007*
12 months after treatment	43.8 ± 5.0	40.3 ± 3.4	0.005*
VD of SCP (ParaFovea)			
Baseline	44.9 ± 4.1	42.4 ± 5.1	0.089
3 months after treatment	44.8 ± 5.0	41.4 ± 4.8	0.017*
12 months after treatment	46.0 ± 5.7	42.4 ± 3.9	0.010*
VD of DCP (Whole Image)			
Baseline	42.9 ± 4.4	41.4 ± 5.2	0.312
3 months after treatment	42.6 ± 5.5	40.7 ± 5.9	0.253
12 months after treatment	45.8 ± 5.0	42.1 ± 4.4	0.011*
VD of DCP (ParaFovea)			
Baseline	44.3 ± 4.9	43.4 ± 5.7	0.561
3 months after treatment	44.0 ± 6.2	42.8 ± 6.1	0.493
12 months after treatment	46.9 ± 5.4	44.2 ± 5.0	0.090
FAZ size			
Baseline	0.33 ± 0.13	0.41 ± 0.22	0.168
3 months after treatment	0.33 ± 0.13	0.42 ± 0.16	0.036*
12 months after treatment	0.34 ± 0.14	0.47 ± 0.18	0.012*
FAZ perimeter			
Baseline	2.35 ± 0.48	2.70 ± 0.79	0.094
3 months after treatment	2.39 ± 0.41	2.76 ± 0.62	0.027*
12 months after treatment	2.30 ± 0.70	2.95 ± 0.72	0.004*

*VD* vessel density; *SCP* superficial capillary plexus; *DCP* deep capillary plexus; *FAZ* foveal avascular zone

*P < 0.05

**Table 3:** Comparison of the change of microvascular parameters measurements in younger and older groups

	Younger group (*n* = 20)	Older group (*n* = 46)	*P*
Delta-VD of SCP			
Whole Image	1.9 (-1.5, 3.3)	-0.6 (-2.2, 2.3)	0.189
ParaFovea	0.2 (-1.5, 3.3)	-0.2 (-2.4, 1.6)	0.261
Delta-VD of DCP			
Whole Image	2.4 (0.1, 6.3)	-0.7 (-3.6, 3.6)	0.037*
ParaFovea	2.3 (-2.0, 5.9)	-0.9 (-3.0, 2.9)	0.056
Delta-FAZ size	0.00 (-0.05, 0.07)	0.04 (0.00, 0.13)	0.061
Delta-FAZ perimeter	0.02 (-0.12, 0.27)	0.16 (-0.01, 0.35)	0.135

Delta = Value at 12 months after treatment - Value before treatment

*VD* vessel density; *SCP* superficial capillary plexus; *DCP* deep capillary plexus; *FAZ* foveal avascular zone

*P < 0.05

**Table 4:** Vision changes after treatment

	Younger Group (*n* = 20)	Older Group (*n* = 46)	*P*
BCVA (LogMAR)			
Baseline	0.63 ± 0.44	0.81 ± 0.36	0.078^a^
12 months after treatment	0.27 ± 0.36	0.51 ± 0.35	0.003^b^*
Vision Improvement Rate (%)^c^	65.6 ± 33.2	40.2 ± 32.6	0.007^a^*

^a^: student *t* test;

^b^: Mann-Whitney *U* test;

^c^: Vision Improvement Rate = (Value at 12 months after treatment - Value at baseline)/Value before treatment

*BCVA* best corrected visual acuity

*P < 0.05

**Table 5:** The correlation between microvasculature parameters change and vision improvement rate all patients

	Vision Improvement Rate	
	*r*	*P*
Delta-VD of SCP		
Whole Image	0.114	0.461
ParaFovea	0.229	0.140
Delta-VD of DCP		
Whole Image	0.359	0.018*
ParaFovea	0.374	0.013*
Delta-FAZ size	-0.218	0.159
Delta-FAZ perimeter	-0.142	0.365

Delta = Value at 12 months after treatment - Value at baseline;

Vision Improvement Rate = (logMAR vision at 12 months after treatment - logMAR vision at baseline)/logMAR vision at baseline;

*VD* vessel density; *SCP* superficial capillary plexus; *DCP* deep capillary plexus; *FAZ* foveal avascular zone

## Discussion

RVO prevalence increases with age, probably due to increased atherosclerosis in elderly patients [6]. The Virchow triad of hypercoagulability, endothelial injury, and stasis of blood flow plays a key role in the process of thrombogenesis in RVO [6]. Atherosclerotic diseases such as hypertension, dyslipidemia, and diabetes are important contributors to thrombogenesis and occur more frequently with aging [6]. However, the pathogenesis and clinical progress of RVO in young patients may be different from in elderly patients [2]. RVO in young patients is closely linked to ocular or systemic diseases such as glaucoma, thrombophilia, autoimmune disease, and oral contraceptive use [2, 6]. It is thought that increased IOP leads to stasis of the retinal vein blood flow at the level of the lamina cribosa, damaging the venous endothelium and predisposing it to thrombosis [7]. In our study, 20 % of young patients had glaucoma, 30 % had traditional risk factors, and 30 % had other systemic disease. No contributing factor was identified in the remaining young patients.

The main vision-threatening complications of RVO include macular edema, retina neovascularization, neovascular glaucoma, and vitreous hemorrhage. Ischemic RVO usually has a poor visual prognosis than non-ischemic one [8]. Previous studies reported better baseline and final visual acuity in younger patients than in older patients with CRVO [2, 6]. Although the initial vision was better in the younger group than the older group in our study, the difference was not statistically significant (*P* = 0.078). However, younger patients had significantly better final vision at 12 months after the first anti-VEGF treatment than older patients. The ocular condition, such as lens opacity, might contribute to this difference. Therefore, we used vision improvement rate as an index in order to avoid the influence caused by age. Even so, younger patients showed better visual improvements compared with older patients.

OCTA enables noninvasive visualization of retinal vasculature and precise assessment of vascular changes at the capillary level [3]. Unlike FFA showing leaking and staining of the lesions, OCTA can capture subtle changes of microvasculature, including neovascular fronds, microaneurysms, non-perfusion area, and other microvascular abnormalities [3, 9]. Additionally, OCTA shows the microvascular changes in both the SCP and DCP and can be used to conduct depth-resolved studies of microcirculation [3, 9]. Shahlaee et al. have reported a negative correlation between age and retinal vascular density in a healthy population [10]. Wakabayashi et al. have reported that eyes with CRVO and BRVO had lower VD in the superficial and deep vascular layers compared to the fellow eye and normal eyes [11].

High intraocular levels of VEGF are thought to contribute to the development of macular edema and progression of ischemia in RVO [12, 13]. Long-term therapy of anti-VEGF injection has been reported to improve, or at least preserve, retinal perfusion in eyes with RVO [14–16]. However, Sellam et al. reported a slight decrease in VD in SCP after anti-VEGF injection in patients with RVO [17]. Spaide showed that anti-VEGF treatment did not change the VD in either superficial or deep capillary plexus in eyes with RVO [18]. In this study, the older group had lower VD than the younger group at baseline, but the difference did not reach the level of significance. The anti-VEGF injections had no significant effect on the VD in the older group at the 12-month follow-up compared with the initial visit. The FAZ size was significantly increased in the older group during the 12-month follow-up. However, there was significantly increased VD in both SCP and DCP in the younger group during the course of follow-up. Younger patients had higher VD in both SCP and DCP, as well as smaller FAZ than the older ones at the 12th month after the first anti-VEGF treatment. The results of studies reporting the effects of anti-VEGF therapy on FAZ area are conflicting in retina vascular disease. Ghasemi Falavarjani et al. reported no statistical difference in FAZ size in the short-term after a single intravitreal anti-VEGF injection in patients with RVO and diabetic macular edema (DME) [19]. Others found no change in FAZ area at 12-month after anti-VEGF treatment in DME patients [20] and RVO patinets [14]. Gill et al. observed a significant FAZ reduction over time after anti-VEGF in DME patients [21]. However, no study above analyzed the impact of anti-VEGF agents on FAZ based on the age of patients. In contrast, we observed enlargement of FAZ during follow-up after treatment in older patients, which is thought to be caused by failure of regeneration of retina microvasculature in this group of patients.

Visual prognosis of RVO usually depends on the initial visual acuity, the extent and the localization of the occlusion, and the retinal perfusion, especially in the macular area [6, 22, 23]. Several studies have shown that final vision was correlated with VD in both the SCP and DCP, and the most significant predictor was vascular perfusion in the DCP [11, 19, 24, 25]. Consistent with previous studies, our study showed a significant correlation between vision improvement and changes in the VD of DCP. Rapid rehabilitation of blood vessels indicated better visual improvements. As previously reported, the DCP is comprised of capillaries with a vortex configuration and drains into large superficial veins [26, 27]. The DCP contains capillaries with higher perfusion pressure and oxygenation, which may be more prominent in protecting the retina from increased venous pressure under RVO. We also compared younger and older patients with regard to changes in retinal perfusion and found that the younger group had more rapid improvement in the VD of DCP. These results indicated that the younger patients with RVO had more rapid rehabilitation of retinal microvasculature after treatment, especially in the DCP, which may lead to better VA improvements. Thus, age is an important factor that may contribute to the retinal blood flow and final vision outcome.

The limitations of this study include its retrospective design, the small number of young patients, and the limited OCTA field of view for analyzing. Loss of follow-up several months after the first anti-VEGF injection due to fast vision recovery of some young patients also generated bias in this study. Some BRVO patients were not enrolled in the study due to lack of macular involvement or loss of follow-up after achieving a satisfied vision, leading to the unnatural rate of BRVO to CRVO patients in this study.

## Conclusions

Age might be an important factor in the prognosis of patients with RVO. Younger patients have more rapid and better reconstruction of retinal perfusion than older patients which can contribute to better final vision.

## Data Availability

The datasets used and analyzed during the current study are available from the corresponding authors on reasonable request.

## Abbreviations

RVO: Retinal vein occlusion
BCVA: Best corrected visual acuity
VD: Vessel density
FAZ: Foveal avascular zone
OCTA: Optical coherence tomography angiography
PAR: projection artifact removal
CRVO: Central RVO
BRVO: Branch RVO
SSADA: Split-spectrum amplitude decorrelation angiography
DCP: Deep capillary plexus
SCP: Superficial capillary plexus
CRT: Central retinal thickness
IOP: Intraocular pressure
FFA: Fundus fluorescein angiography
NPAs: Nonperfused areas
DA: Disk area

## References

[1] Rogers S, McIntosh RL, Cheung N, Lim L, Wang JJ, Mitchell P, et al. The prevalence of retinal vein occlusion: pooled data from population studies from the United States, Europe, Asia, and Australia. Ophthalmology. 2010;117(2):313–9 e1. 10.1016/j.ophtha.2009.07.017 PMC2945292 20022117

[2] Rothman AL, Thomas AS, Khan K, Fekrat S, CENTRAL RETINAL VEIN OCCLUSION (2019). IN YOUNG INDIVIDUALS: A Comparison of Risk Factors and Clinical Outcomes. Retina.

[3] Tsai G, Banaee T, Conti FF, Singh RP (2018). Optical Coherence Tomography Angiography in Eyes with Retinal Vein Occlusion. Journal of ophthalmic & vision research.

[4] Zhu T, Ma J, Li J, Dai X, Ye P, Su Z (2019). Multifractal and lacunarity analyses of microvascular morphology in eyes with diabetic retinopathy: A projection artifact resolved optical coherence tomography angiography study. Microcirculation.

[5] Zhu TP, Li EH, Li JY, Dai XZ, Zhang HN, Chen BB (2020). Comparison of Projection-Resolved Optical Coherence Tomography Angiography-Based Metrics for the Early Detection of Retinal Microvascular Impairments in Diabetes Mellitus. Retina.

[6] Garcia-Horton A, Al-Ani F, Lazo-Langner A (2016). Retinal vein thrombosis: The Internist’s role in the etiologic and therapeutic management. Thromb Res.

[7] Marcucci R, Sofi F, Grifoni E, Sodi A, Prisco D (2011). Retinal vein occlusions: a review for the internist. Intern Emerg Med.

[8] Rogers SL, McIntosh RL, Lim L, Mitchell P, Cheung N, Kowalski JW (2010). Natural history of branch retinal vein occlusion: an evidence-based systematic review. Ophthalmology.

[9] Chung CY, Tang HHY, Li SH, Li KKW (2018). Differential microvascular assessment of retinal vein occlusion with coherence tomography angiography and fluorescein angiography: a blinded comparative study. International ophthalmology.

[10] Shahlaee A, Samara WA, Hsu J, Say EA, Khan MA, Sridhar J (2016). In Vivo Assessment of Macular Vascular Density in Healthy Human Eyes Using Optical Coherence Tomography Angiography. American journal of ophthalmology.

[11] Wakabayashi T, Sato T, Hara-Ueno C, Fukushima Y, Sayanagi K, Shiraki N (2017). Retinal Microvasculature and Visual Acuity in Eyes With Branch Retinal Vein Occlusion: Imaging Analysis by Optical Coherence Tomography Angiography. Investigative ophthalmology & visual science.

[12] Campochiaro PA, Bhisitkul RB, Shapiro H, Rubio RG (2013). Vascular endothelial growth factor promotes progressive retinal nonperfusion in patients with retinal vein occlusion. Ophthalmology.

[13] Sophie R, Hafiz G, Scott AW, Zimmer-Galler I, Nguyen QD, Ying H (2013). Long-term outcomes in ranibizumab-treated patients with retinal vein occlusion; the role of progression of retinal nonperfusion. American journal of ophthalmology.

[14] Winegarner A, Wakabayashi T, Fukushima Y, Sato T, Hara-Ueno C, Busch C (2018). Changes in Retinal Microvasculature and Visual Acuity After Antivascular Endothelial Growth Factor Therapy in Retinal Vein Occlusion. Investigative ophthalmology & visual science.

[15] Mir TA, Kherani S, Hafiz G, Scott AW, Zimmer-Galler I, Wenick AS (2016). Changes in Retinal Nonperfusion Associated with Suppression of Vascular Endothelial Growth Factor in Retinal Vein Occlusion. Ophthalmology.

[16] Suzuki N, Hirano Y, Tomiyasu T, Esaki Y, Uemura A, Yasukawa T (2016). Retinal Hemodynamics Seen on Optical Coherence Tomography Angiography Before and After Treatment of Retinal Vein Occlusion. Investigative ophthalmology & visual science.

[17] Sellam A, Glacet-Bernard A, Coscas F, Miere A, Coscas G, Souied EH, QUALITATIVE AND QUANTITATIVE FOLLOW-UP USING, OPTICAL COHERENCE TOMOGRAPHY ANGIOGRAPHY OF RETINAL VEIN OCCLUSION TREATED WITH ANTI-VEGF (2017). Optical Coherence Tomography Angiography Follow-up of Retinal Vein Occlusion. Retina.

[18] Spaide RF (2016). Volume-Rendered Optical Coherence Tomography of Retinal Vein Occlusion Pilot Study. American journal of ophthalmology.

[19] Casselholmde Salles M, Kvanta A, Amren U, Epstein D (2016). Optical Coherence Tomography Angiography in Central Retinal Vein Occlusion: Correlation Between the Foveal Avascular Zone and Visual Acuity. Investigative ophthalmology & visual science.

[20] Conti FF, Song W, Rodrigues EB, Singh RP (2019). Changes in retinal and choriocapillaris density in diabetic patients receiving anti-vascular endothelial growth factor treatment using optical coherence tomography angiography. International journal of retina and vitreous.

[21] Gill A, Cole ED, Novais EA, Louzada RN, de Carlo T, Duker JS (2017). Visualization of changes in the foveal avascular zone in both observed and treated diabetic macular edema using optical coherence tomography angiography. International journal of retina and vitreous.

[22] Lang GE, Lang SJ (2018). [Retinal Vein Occlusions]. Klinische Monatsblatter fur Augenheilkunde.

[23] Hayreh SS, Podhajsky PA, Zimmerman MB (2011). Natural history of visual outcome in central retinal vein occlusion. Ophthalmology.

[24] Seknazi D, Coscas F, Sellam A, Rouimi F, Coscas G, Souied EH (2018). OPTICAL COHERENCE TOMOGRAPHY ANGIOGRAPHY IN RETINAL VEIN OCCLUSION: Correlations Between Macular Vascular Density, Visual Acuity, and Peripheral Nonperfusion Area on Fluorescein Angiography. Retina.

[25] Samara WA, Say EA, Khoo CT, Higgins TP, Magrath G, Ferenczy S (2015). Correlation of Foveal Avascular Zone Size with Foveal Morphology in Normal Eyes Using Optical Coherence Tomography Angiography. Retina.

[26] Bonnin S, Mane V, Couturier A, Julien M, Paques M, Tadayoni R (2015). New Insight into the Macular Deep Vascular Plexus Imaged by Optical Coherence Tomography Angiography. Retina.

[27] Paques M, Tadayoni R, Sercombe R, Laurent P, Genevois O, Gaudric A (2003). Structural and hemodynamic analysis of the mouse retinal microcirculation. Investigative ophthalmology & visual science.

